# Modelled broad-scale shifts on seafloor ecosystem functioning due to microplastic impacts on bioturbation

**DOI:** 10.1038/s41598-023-44425-8

**Published:** 2023-10-10

**Authors:** Yuxi You, Alice Della Penna, Simon Francis Thrush

**Affiliations:** 1 Institute of Marine Science, The University of Auckland, Auckland, 1010 New Zealand; 2 School of Biology Science, The University of Auckland, Auckland, 1010 New Zealand

**Keywords:** Environmental impact, Ecological modelling

## Abstract

Bioturbating species play an essential role in regulating nutrient cycling in marine sediments, but their interaction with microplastics (MP) remains poorly understood. Here we investigated the linkage between MP and ecosystem functioning using experimental observations of luminophore distribution in the sediment to parametrize bioturbation coefficients (D_b_). this information as fed into a simplified transport-reaction model, allowing us to upscale our experimental results. We found that the composition of bioturbators modulated shifts in the ecosystem functioning under microplastic stress. Maldanid worms (*Macroclymenella stewartensis)*, functionally deep burrowing and upward-conveyor belt feeders, became less active. The D_b_ of *M. stewartensis* reduced by 25% with the addition of 0.002 g MP cm^−2^ at surface sediment, causing accumulation of organic matter in the oxic sediment zone and stimulating aerobic respiration by 18%. In contract, the tellinid bivalve *Macomona liliana,* functionally a surface -deposit feeder that excretes at depth*,* maintained particle mixing behaviour in MP-contaminated systems. This study provides a mechanistic insight into the impacts of MP and indicates that the functional role of bioturbating species should be involved in assessing the global impact of MP. The model allowed us to understand the broad-scale impact of MP on seafloor habitat.

## Introduction

Plastics of micro- and nano-size are the most hazardous and wide-spread plastic pollution^1–3^, but their ecological impacts involve complex interactions among environmental components that are largely unknown ^4, 5^. Most microplastics (MP, dia. < 0.5 cm) end up on the seafloor, particularly in coastal sediments^6–9^. Plastic particles deposited in sediment habitats are threatening benthic species. For example, the polychaete *Arenicola marina* suffered a 50% loss of energy reserves with reduced feeding rates when they ingested unplasticized polyvinylchloride (UPVC) particles^10^. Green et al. (2016) demonstrated that after one-month exposure *A. marina’s* burrowing capacity decreased with increasing dosages of MP (from 0.02%, 0.2%, 2% by wet weight sediment)^11^. The deposit-feeding bivalve *Macomona liliana* lost burrow capacity after 31-day exposure to polyethylene terephthalate microplastics (PET) (1% by sediment weight)^12^_._ Recent studies found sediment dwellers (e.g., the deposit feeding bivalve *Limecola balthica*) avoids the MP by penetrating deeper to the sediment and reduces their food intakes^13^. These effects on worm and bivalve species lead to changes in their behaviours and potential shifts in ecosystem function roles, notably in bioturbation.

Bioturbation is referred as the movement of sediment particles and porewater by animals: it plays a critical functional role of benthic macrofauna mediating the ecosystem responses and enhances the cycling of carbon and nitrogen in marine environments^14–17^. Bioturbation can take many forms depending on animal body-size, density, feeding and burrowing strategies. These factors affect how animals redistribute particles from the sediment surface to different depths^18, 19^, influence the sediment erodibility^20, 21^, determine the heterogeneity of biogenic habitats, and maintain the resilience of ecosystem functions^22, 23^. Bioturbation modes also influence how MP transport in the sediments. The conveyor-belt feeders that can transport particles in both up and downward directions might induce a deep penetration of MP in the sediment, while biodiffusors with strict upward conveying mode retain less MP in the deep zone^24^. However, the cumulative effects of MP pollution on bioturbation will depend on the sensitivity of individual species and the specific role they play in the sediment. The loss of large macrofauna and their functional roles can lead to a cascading effect on the ecosystem functioning^25–27^; therefore, the potential for broad-scale shifts in ecosystem functions due to the impact of MP on the macrofauna playing a role as bioturbator needs to be assessed^28, 29^.

Here, we upscale the impacts of MP to an ecosystem level by reparametrizing the bioturbation coefficient D_b_. D_b_ is derived from the biological mixing patterns that reflect the response of bioturbators to their environmental conditions (e.g., contamination, sedimentation, food availability)^16, 30^. For example, in petrol-contaminated sediments, the benthic infauna community contributed to a peak accumulation of particles 2–4 cm sediment depth^31^. Similarly, the polychaete (*Perinereis adbuhitensis*) lost its deep transporting ability with particle penetration to a depth of 6–8 cm reduced with the increasing concentrations of cadmium and copper^32^. Such observed changes in particle transport profiles are the indicator of pollution stress on infaunal groups.

In transport-reaction models ^17, 33, 34^, the bioturbation coefficient (D_b_) represents the macrofaunal function role in reworking sediment particles and transferring organic matter (OM) to the microbial community in the sediment as a critical step for OM degradation. The penetrated OM stimulates microbial activities at different sediment zones e.g., aerobic mineralization and denitrification ^35–37^. In a simplified sediment system, aerobic mineralization takes place in the oxic zone^34^. The anoxic zone is the central place for multiple reduction processes such as NO_3_^-^ being reduced to NH_4_^+^ or denitrifying to N_2_^38^; the reduced products from anaerobic processes in turn also enhance denitrification^17^. Potentially, the bioturbation coefficient D_b_ could mediate the impact of MP to ecosystem functions and allow observations of how MP changes D_b_ to be upscaled to assess broadscale consequences of MP pollution in marine seafloors.

**Figure 1:**
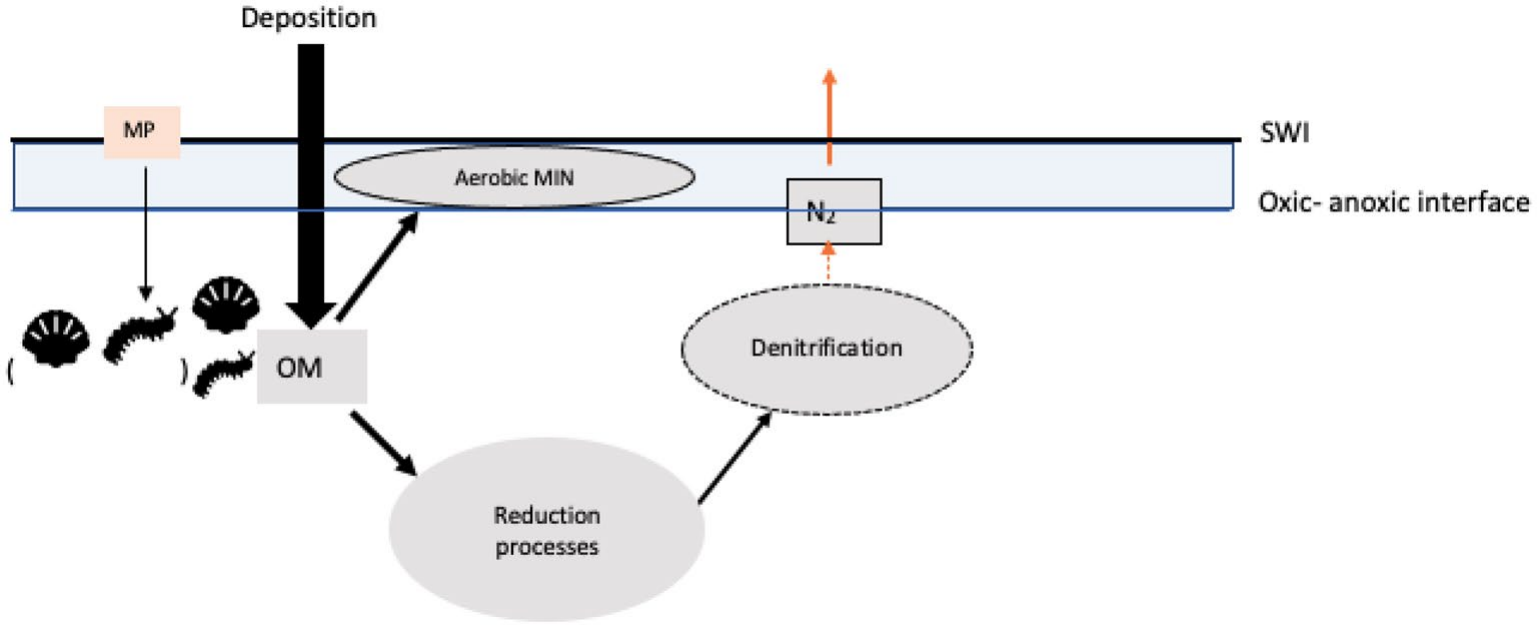
Conceptual simplified diagram of MP interacting with bioturbation and associated processes from OM degradation. *Aerobic MIN* aerobic mineralization; N_2_ production is the outcome from denitrification processes (orange arrow). Bivalve and worm symbols imply sediment reworking by these bioturbating species. This diagram is modified from conceptual diagram in Middelburg (2019)^17^.

In this study, we measured D_b_ in a laboratory experiment investigating the impacts of MP on two large and functionally important species, the maldanid polychaete *Macroclymenella stewartensis* and tellinid bivalve *Macomona liliana*. D_b_ measurements made under different MP concentrations were then used to parameterize a simplified transport-reaction model^34^ (Table S2, supplementary). We choose this model because it provides estimates of organic matter (OM) fluxes down the sediment column. D_b_ is a key driver of the redistribution of OM influxes and thus the portion of aerobic mineralization in the oxic sediment^34^. This model assumes that, in the oxic zone, the penetrated OM induces equivalent oxygen demands for the complete aerobic degradation. In the anoxic zone, the penetrated OM participates in denitrification processes, and multiple anaerobic processes deliver the reduced components (e.g., NH_4_^+^, Fe^2+^, Mn^2+^, H_2_S) to enhance the N-cycling^17, 39^. The difference between OM inputs and aerobic mineralization in this virtual ecosystem provides a proxy for reduction processes. Denitrification consumes energy from penetrated OM to regenerate N_2_^17^. In the context of this study, N_2_ production is a proxy for reduction processes that omits the complexity of multiple degradation pathways, and it is the source of the N_2_ released from sediment–water interface (SWI) (Fig. 1).

## Results

### Bioturbation coefficient D_b_ vary in response to changes in MP concentration

**Figure 2:**
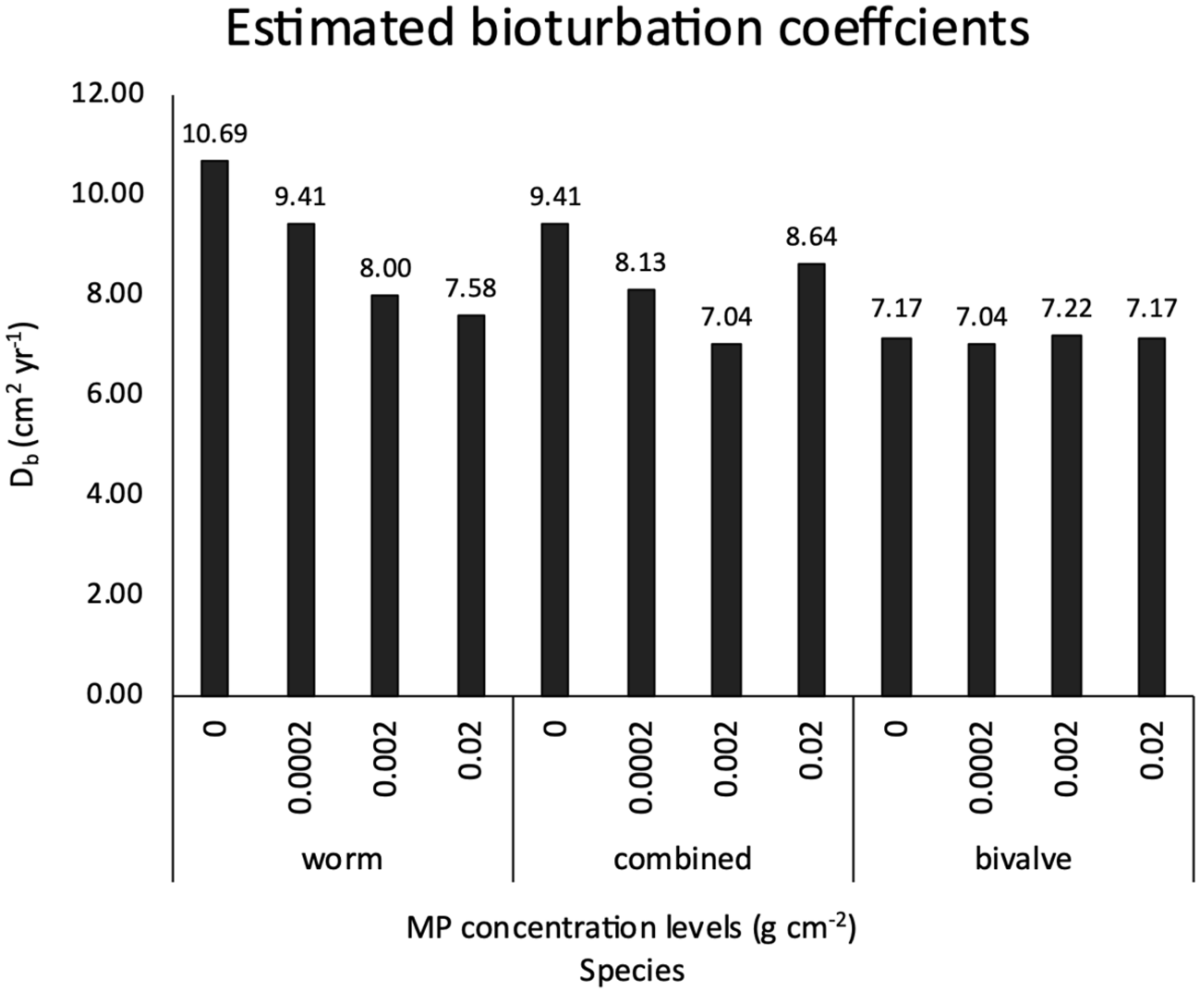
Estimated bioturbation coeffcient D_b_ (cm^2^ year^−1^) of worm, combined and bivalve groups in MP treatments.

In treatments hosting only worms or the combined two species, decreases of D_b_ compared to the control were 25% with 0.002 g cm^−2^ of MP (Fig. 2; Table 1). The highest D_b_ value (10.69cm^2^ year^−1^) was measured in the worm groups without MP and changes of D_b_ compared to the control was almost -30% with high concentration level of MP (0.02 g cm^−2^) (Table 1). Bivalve groups had a relatively stable particle mixing intensity (7.04–7.22 cm^2^ year^−1^) in MP treatments. The difference between worm and bivalve groups on D_b_ values decreased (7.58 cm^2^ year^–1^ vs. 7.17 cm^2^ year^−1^) when exposed to a higher concentration of MP (0.02 g cm^−2^).

**Table 1:** Percentage changes of D_b_ in MP treatment (increased concentration from 0.0002 to 0.02 g cm^−2^) compared to that in the control.

Increases of MP concentrations (g cm^-2^)	Changes (%) of D_b_ compared to the control		
	Worm (%)	Combined (%)	Bivalve (%)
0.0002	−12	−14	−2
0.002	−25	−25	1
0.02	−29	−8	0

**Figure 3:**
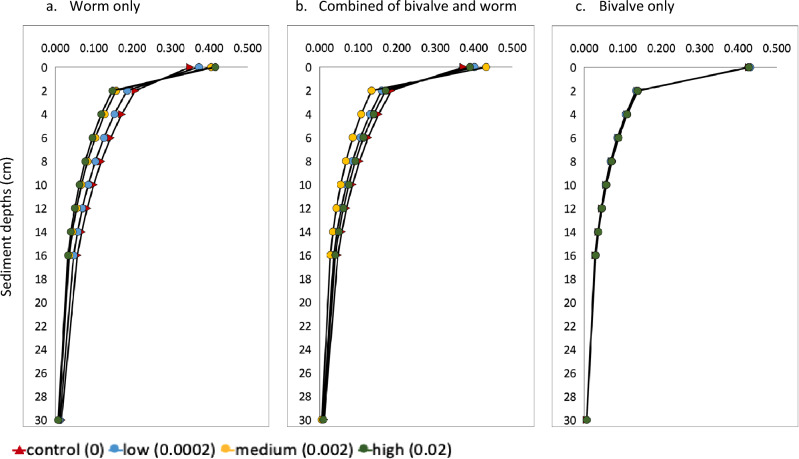
Simulated OM concentrations along sediment depth from species groups (**a**. worm, **b**. combined of bivalve and worm, **c**. bivalve) with concentration levels of MP: 0, 0002, 0.002, 0.02 g cm^−2^. Note that in (**c**) the four lines overlap.

Concentrations of OM penetrated along sediment depths were determined by D_b_ from bivalve, combined and worm groups separately (Fig. 3). In worm groups, MP treatments had more OM depositing in the sediment surface (0–2 cm). The group with combined of bivalve and worm had a similar trend. A convergence of OM concentration profiles from bivalve group shows there was no change in OM penetration profiles after adding MP.

### Ecosystem functioning in MP-bioturbator groups

**Figure 4:**
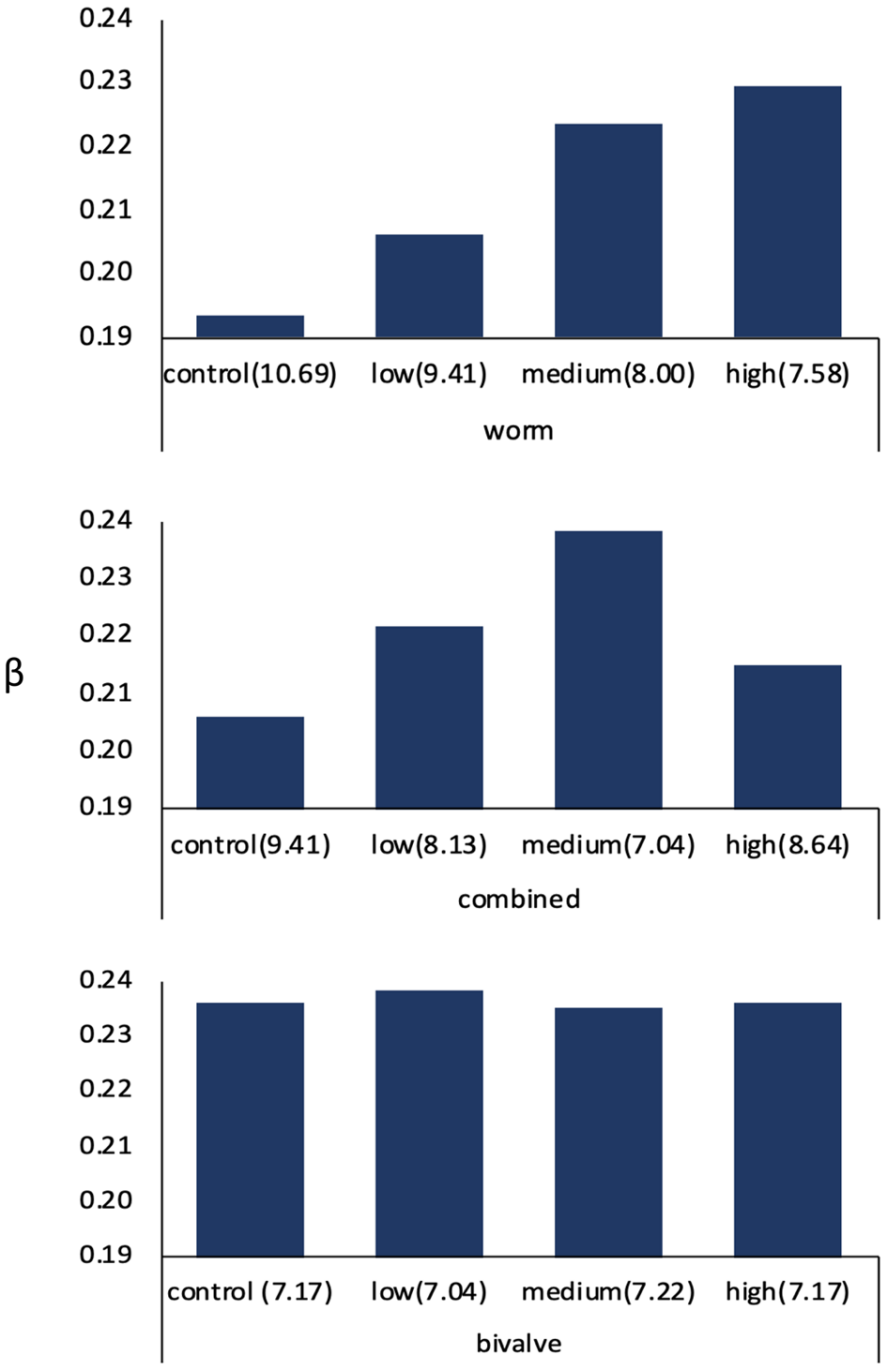
Fraction of aerobic mineralization β at oxic zone in worm, combined and bivalve groups from MP treatments at control, low medium and high levels (D_b_ values in parenthesis).

Fraction of aerobic mineralization (β) accounting to total OM degradation varied with D_b_ in the worm-only and combined groups, and this induced changes on oxygen consumption and nitrogen production (Fig. 4; Table 2). In the worm groups, aerobic mineralization fraction increased when D_b_ decreased: the percentage of change of O_2_ consumption rates was from 6.58 to 18.73% when MP concentration increased from 0.0002 to 0.02 g cm^−2^ (Fig. 4; Table 2). N_2_ production compared to the control decreased from 1.58 to 4.49% at the expense of increased aerobic mineralization. A similar, but weaker trend was in the combined groups: when MP concentration reached 0.002 g cm^−2^, the O_2_ consumption rates compared to the control increased by 15.66%. The fraction of aerobic mineralization β, oxygen consumption rates F__o2_ and estimated N_2_ production R_(N)_ were invariant in bivalve groups when MP concentration increased.

**Table 2:** Percentage changes compared to control on oxygen consumption rates (F__o2_) and estimated N_2_ production (values in square brackets) with increases of MP concentrations.

Increases of MP concentrations (g cm^−2^)	Changes (%) of oxygen consumption rates (F__o2_) and [estimated N_2_ production (R_(N)_)] compared to the control		
	Worm, % [%]	Combined, % [%]	Bivalve, % [%]
0.0002 (low)	6.58 [−1.58]	7.58 [−1.97]	0.97 [−0.30]
0.002 (medium)	15.64 [−3.75]	15.66 [−4.07]	−0.31 [0.10]
0.02 (high)	18.73 [−4.49]	4.09 [−1.14]	0.00 [0.00]

## Discussion

### Bioturbation coefficients in MP-contaminated system

We expect that the functional traits of different species, such as the feeding and movement of worms and bivalves determine the responses of the sediment ecosystem to MP pollution. In our study, the highest D_b_ was observed in experiments occupied by worms without MP and this value decreased as MP concentrations increased, whereas the D_b_ of bivalves was relatively stable. As a tube builder, the maldanid polychaete *M. stewartensis* feeds head-down around 10 cm depth in the sediment and excretes faeces on the sediment surface^40^. This burrowing mode enlarges turnover areas through sediment columns, increasing the oxygen penetration depth and subducting more labile OM to the deep zone ^41, 42^. This behaviour can explain higher bioturbation coefficients in the worm groups compared to the bivalve ones. Exposure to MP increases the risk of worms ingesting MP, and this may cause health issues including gut inflammation, reduction of their ingestion rates and energy reserves, suppression of burrowing activities, and limitation to reproduction^10, 11^. The D_b_ values of worms decreased to a level similar to the bivalve’s when MP concentration was increased. A reduced bioturbation rate means less sediment turnover scales and particle mixing^43^. While our results imply potential toxic effects of MP on maldanid burrowing behaviour, this is not apparent for the deposit-feeding bivalve (*M. liliana*) which uses a long siphon to capture the food particles at the sediment surface^44^. Previous studies have shown that *M. liliana* are impacted by MP with reduced reburial rates^12, 45^ which seem to contrast our finding in terms of bioturbation. However, in this study we focused on particle movement to calculate D_b_, whereas the role of *M. liliana* in regulating the nutrient cycling may be more strongly linked to porewater advection and redox oscillations^46^. *M. liliana* periodically pressures overlying fluids and oxygenized-water to the burrowed zone during feeding period^47^. Although the particle reworking intensity of the bivalve group in MP treatments remains stable, it might be because the parameter D_b_ has limitations in explaining these fluid oscillation behaviours.

MP weakened the particle mixing intensity by maldanid worm and reworking behaviours of tellinid bivalve might dominate the system when MP concentration increased. D_b_ values from worm groups decreased by the increased concentration of MP; the values from bivalve and combined groups converged to a similar level (7.22, 7.04 cm^2^ year^−1^) when MP concentration reached 0.002 g cm^−2^. Chemical pollution has been reported to reduce the bioturbation potential as a result of the losing species diversity and reduced community biomass^48^. Large and functional important species have dominant impacts on particle redistribution and nutrient cycling as well as the community structure, which is more influential than species diversity^25, 27, 49^. Bioturbating species take different strategies to adapt to environment stresses (e.g., marine heatwaves, acidification, nutrient loading), with the cascading-effect on ecosystem functioning depends on the functional traits of key species^50–52^. Therefore, feedback from the ecosystem (e.g., nutrient fluxes and oxygen consumption) associated with sediment reworking will depend on the responses from relative dominant species to MP and the specific mechanisms by which they fulfil their individual and collective functional roles. In this study, changes of D_b_ reflect a potentially different impact of MP on the sediment reworking in the areas dominant with maldanid worm *M. stewartensis* and tellinid bivalve *M. liliana* separately, as well as their transition zones (co-occur of these species) in the seafloor habitats. The bioturbation of *M. liliana* and *M. stewartensis* creates distinct microtopographic features on the sediment surface that influence the nutrient fluxes^53^. In MP-contaminated habitats, the maldanid worm can loss advantage in deep-particle mixing and maintaining the nutrient cycling. Functional traits of *M. liliana* associated with sediment reworking and generation of porewater pressure gradients will lead the ecosystem processes when two species co-occur, and the habitats becomes homogenized regarding the loss of worm’s functional roles. This is likely to happen in the natural habitat when the concentration of MP continues to increase. Currently, there is no consistent way to measure MP concentration, and differences from areal concentrations to mass-based concentrations are common. MP concentrations in the field are often variable, sites with concentration spikes are associated with the in-situ breakdown of larger plastic items^9, 54, 55^. The range of MP used in our treatments reflects both the variation between sites but also the potential extreme values within sites associated with spatial and temporal dynamics^56–58^. Even MP increasing to 0.002 g cm^−2^ is concerning because the similar contamination level was found in field studies^59^.

### Ecological consequences from MP-contaminated system

**Figure 5:**
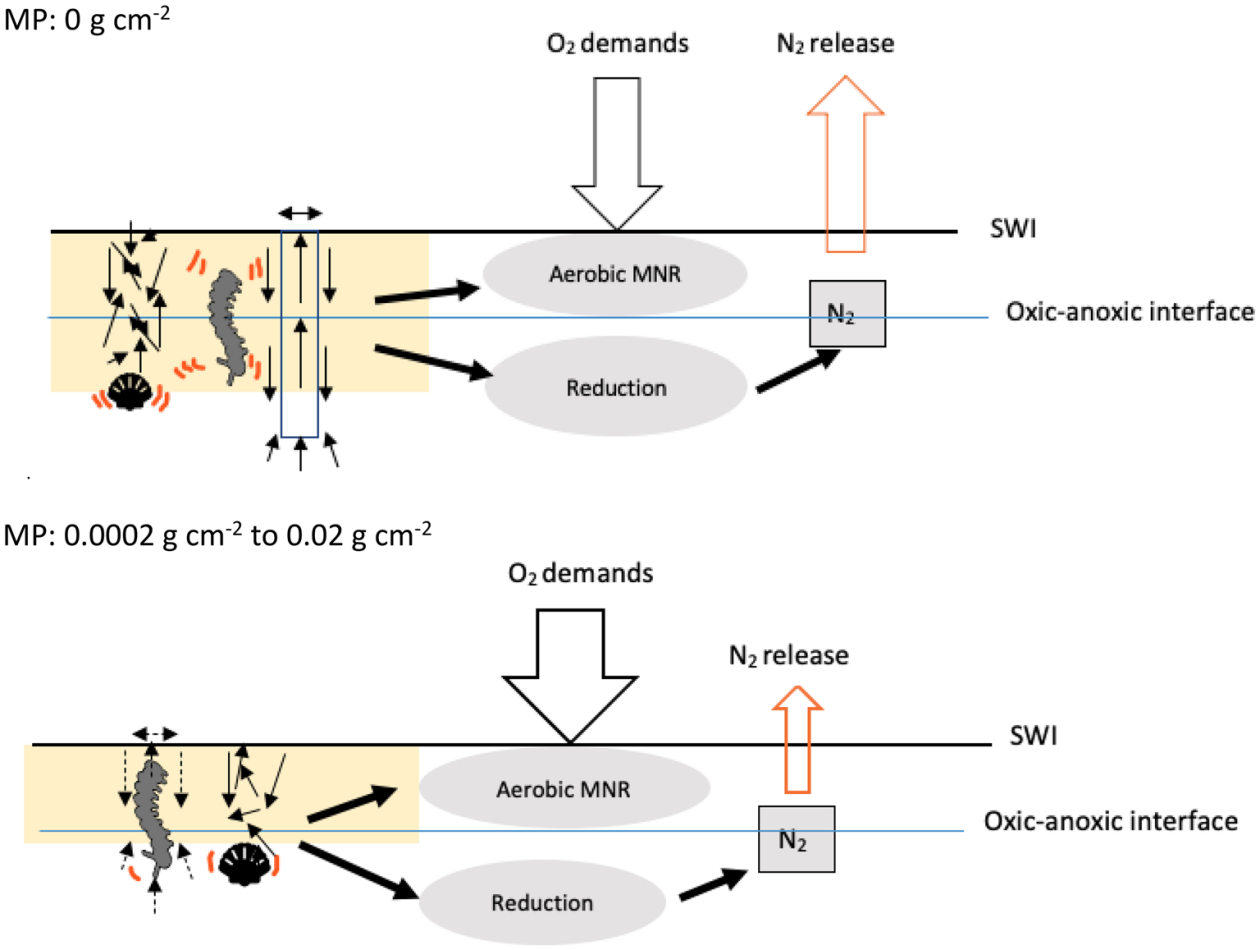
Schematic representation of ecosystem functions in MP- and control seafloor based on the study outputs (modified from Middelburg, 2019^17^). Yellow zone: OM penetration pattern. *SW* sediment–water interface, *aerobic MNR* aerobic mineralization, Reduction: reduction processes at anoxic zone: Bivalve and worm symbols: burrowing patterns of maldanid worm *Macroclymenella stewartensis* and tellinid bivalve *Macomona liliana* (modified from Schenone et al., 2019^40^). N_2_: nitrogen production from reduction processes that potentially can release at SWI.

Our measurement of D_b_ under different MP loads combined with the application of the transport-reaction model indicates that in plastic polluted sediments, less organic matter (OM) is subducted deep into the sediment resulting in higher oxygen consumption in the sediment. The associated effects on ecosystem functioning with bioturbations are summarized in Fig. 5.

The shallow penetration of OM stimulates microbial activities in the oxic zone with more oxygen demands for the respiration. Increasing oxygen demands in MP-contaminated sediments were reported in previous laboratory tests^13^, and was associated with the reduced burrowing capacity of worms and bivalves with different functional traits to our study species (i.e., *Arenicola marina*, *Cerastoderma glaucum*)^11, 13^. The observation from a real-world experiment reveals that mollusc abundance alone cannot explain the increasing oxygen demands in the dark (aerobic respiration), and the linkage of macrofauna and sediment oxygen consumption can be broken with the contamination of fibric microplastic^60^. Our study provides a mechanistic insight into this phenomenon: aerobic-microbial activities consuming OM at the sediment surface may overweigh macrofauna’s mediating effects on microbial activities through oxic-anoxic sediment zones. The OM-enriched sediment surface often needs higher oxygen demands and it causes a reduced oxygen penetration depth^61, 62^. The long-term persistence of this event, in turn, causes the hypoxia stress on macrofauna assemblages and increases the risk of eutrophication ^63, 64^. In the intertidal areas, low-density and the removal of large bioturbators (e.g., *M. liliana*, *M. stewartensis* and *A. marina*) often lead to a lower consumption of carbon sourced from microphytobenthos (MPB) on sediment surface^65, 66^. In our study, the shallow penetration of OM also indicates few labile OM resources (e.g., MPB) are transported to the sediment subsurface and incorporated to the nutrient cycling. The composition of MPB shifts the trophic linkage between MPB and macrofauna breaks: macrofauna grazing on microphytes provides nutrients (e.g., ammoniacal nitrogen) to maintain the MPB standing stock^49^. However, MP might break this linkage with increasing cyanobacteria biomass in the MPB community, which changes the nutrient cycling in the sediment^45^. The effect of MP is concerning in these scenarios because the biogeochemical processes that rely on particle mixing by large macrofauna will need a longer time to recover, either as the toxicity of the plastic decreases or as more tolerant species with similar functional traits replace the more sensitive species ^67^.

Ecological consequences of MP are context-dependent on the species composition and MP properties (e.g., concentrations); therefore, the outputs should be carefully interpreted due to the simplification of the model. Firstly, the parameter D_b_ may be sensitive to changes in the worm’s activities and deep particle mixing patterns, but it does not represent the changes on bivalve’s fluid oscillation. The hydraulic activities extend the oxic zones and mediate the microbial activities periodically^47, 68, 69^, and this needs to be involved in the further assessment. Secondly, we have not included responses from microbial community and microphytobenthos to MP. Previous studies have shown MP triggering microbial aggregation^70, 71^, shifting the microbial compositions ^72^ and increasing cyanobacteria biomass at the sediment surface ^45^. While these effects may be most pronounced in shallow photic sediments, these are common in harbours and estuaries that often exhibit high MP concentrations. We expect that excluding these components might deviate the prediction from the observation in a real-world experiment. For example, the growth of the cyanobacteria at the photic sediment surface will reduce the downward diffusion of O_2_ fluxes, and O_2_ consumption decreases when gross photosynthesis increases^73^. In this circumstance, the observed O_2_ consumption rate from MP treatments can be a net effect of increased aerobic respiration induced by OM accumulation and gross photosynthesis caused by cyanobacteria. Thirdly, the model assumption of complete degradation of OM in a virtual semi-closed sediment zone^34^ leads to the analytical estimation of O_2_ consumption and N_2_ production via feedbacks to metabolic resource redistribution that is tuned by parameter D_b_. Although N_2_ production in MP-treatments decreased by a small percentage owing to increased aerobic mineralization in worm and combined groups; a weakened nitrogen cycling is likely a result of a chains-reaction when macrofauna loses its functional role in transporting labile OM and regulating microbial activities. This study has not included multiple limiting factors (e.g., OM quality, NO_3_^-^ concentration) in N-cycling, as well as the complexity of pathways of denitrification, including directly denitrifying NO_3_^-^ in overlying water and coupling of nitrification and denitrification processes at an oxic-anoxic sediment interface^74^. MP as synthetic polymers potentially participates in OM degradation^75^, and some materials (PLA) can serve as carbon sources which stimulate both nitrification and denitrification rates^72^. In a eutrophic system, OM (quality and quantity) constrains denitrification when overlying water provides sufficient NO_3_^−^; in a low-nutrient system, the NO_3_^-^ supply from nitrification is the limiting factor for denitrification in the sediment^74^. In this case, we predicted that when the bioturbation rate decreased because of MP, O_2_ demands increased because of OM accumulation. If this phenomenon co-occurs with decreased O_2_ penetration depth, the nitrification can stop due to the limited O_2_ supply, and this decouples nitrification from denitrification in the sediment. These undefined relationships increase the difficulty in predicting MP’s impacts on ecosystem functioning. The relationship between MP and bioturbation needs more empirical data to confirm before upscaling to a global-scale model^76–78^.

## Conclusion

Our study highlights that the impact of MP on bioturbation can result in broad-scale shifts in ecosystem functioning. These effects are the result of interaction between functional traits of bioturbating species and concentration of MP. The strongest effects on function were driven by changes in the deep-burrowing behaviour of maldanid worms. This result indicates that substantial effects of MP pollution on ecosystem function may be more evident in areas dominated by large deep burrowing species rather than in more degraded ecosystems dominated by other smaller macrofauna^25, 49^. The differences in our estimates of D_b_ from the maldanid worm and the tellinid bivalve highlight the importance of considering the specific mechanisms of bioturbation and how they can be incorporated in biogeochemical models. Linking laboratory observation to the numerical model allows us to estimate the consequences at the ecosystem level of MP contamination. Future studies can expand on both the range of functional traits of key species and consider community level effects as well as considering the hydraulic activities of macrofauna alongside particle transport.

## Methods

### Measuring D_b_ under different MP loads

#### Sediment and animal collection

Sediment and animals, the tellinid bivalve *Macomona liliana*, and the maldanid polychaete *Macroclymenella stewartensis,* were collected during low tide from Whāngateau estuary (36°18′52.37" S, 174°46′17.09" E), New Zealand. Sediments (grain size: 164.94 ± 1.58 μm mean ± standard deviation, fine sand fraction: 55.5%, mud content:11.4%) were sieved through 500 μm mesh a to remove t macrofauna. Bivalve *M. liliana* (minimum shell length: 3.8 cm, maximum shell length: 4cm) and worm *M. stewartensis* (body length: ~ 10 cm) were hand-collected from their habitats. The estimated wet weights for bivalve and worm at these sizes were 2.3 g and 0.2 g, respectively ^40^.

#### MP preparation

Prewashed polypropylene plastic pellets (PP, diameter: 4 mm, LINGS limited, China) were frozen for 2 weeks at −80 °C to embrittle the PP particles. The frozen raw materials were ground using a coffee mill (Coffee tech Limited, New Zealand). MP particles (hereafter: MP) were sieved through 500 μm mesh to control the size of MP (diameter < 500 μm).

#### Preincubation

Before being combined with target species, MP were introduced to the top 1 cm surface sediment and incubated from 30th May to 17th, June, 2022. This step is designed to reduce the resuspension of MP. A 2-week incubation allows microalgae to cover the surface of MP ^79^. This bio-stabilization (e.g., growth of biofilm and interact with microalgae) on the sediment bed is the precondition for MP deposition in the sediment ^6^.

Preserved MP and surface sediment were homogenized and incubated in individual containers (1cm depth, surface areas: 727.8 cm^2^). We created a gradient in MP concentrations with values of 0.02, 0.002, 0.0002 g cm^−2^ by surface areas of sediment. Sediments without plastic addition were incubated as a control. Each control and MP treatment was replicated three times, with three blocks of sediment allocated in each container. These preincubated sediments were set up at temperature at ~ 16 °C, and topped up with filtered, clean seawater. A gentle inflow rate ~ 35 ml min^−1^ was set to limit resuspension in the sediment columns. All MP treatments were randomly layout under four double Aqua One Reflector Fluroglow T8 (40 W) sunlight tubes hung 55 cm above the water surface, and set on a 12 h light/dark cycle. The photosynthetic photon flux density (PAR, waveband 400–700 nm) on the surface of incubated water was measured by Li-Cor LI-190R quantum sensor coupled with a Li-Cor data-logger (Li-Cor, USA), with avg. 165.42 ± SD 5.60 μmol photons m^−2^ s^−1^. External light was excluded by a blackout curtain.

#### Main incubation

We measured the sediment reworking profiles from incubations with worms, bivalves, and a combination of the two in MP-contaminated sediment. A total of 36 cylindric buckets (volume: 5 L, height: 24.5 cm) were submerged in the filtered water flow for at least 3 weeks to reduce the release of the plasticizers. All buckets were filled with 11cm clean sieved-sediment and topped with 1 cm surface sediment (surface areas: 240.6 cm^2^) from the preincubation step. These sediments were settled for 24 h and placed in a bath of sand-filtered seawater (salinity: 35.5, temperature: 16 °C, flow rate: ~ 80 ml min^−1^_,_ water depth: 13 cm). Healthy worms and bivalves were placed on the surface sediment and allowed to burrow into the sediment and acclimate for 24 h. The main incubation started with no worms or bivalves on the sediment surface. A total of 34 g of luminophores (florescence painted natural sands, grain size diameter: 149.15 μm ± 0.51 mean ± standard deviation) were evenly spread through the water column to cover the surface sediment at a density of 0.14 g luminophores cm^−2^ in the top 1 cm sediment. A control (0 g MP cm^−2^) and three MP concentration levels (g cm^−2^ surface areas of 1cm depth wet sediment) were used: (1) low concentration (0.0002 g cm^−2^), (2) medium concentration (0.002 g cm^−2^) and (3) high concentration (0.02 g cm^−2^). These were crossed with three animal treatments (a) two *M. liliana* (‘bivalve’), (b) two *M. stewartensis* (‘worm’) and (c) one *M. liliana* with one *M. stewartensis* (‘combined’). The density of two species in each treatment was 83.3 ind. m^−2^ and it is within the ranges of natural density in the habitat ^80^. The two species naturally co-occur in New Zealand intertidal soft sediment^40^. These treatments were replicated three times and incubated from 18th June to 12th July in Leigh marine laboratory, The University of Auckland, New Zealand. Incubation buckets were randomly distributed under the same light condition as that in the preincubation step.

### Luminophore—sediment sampling

Three sediment cores (10 cm depths, diameter: 2.7 cm) were collected from each bucket. At the end of incubation, the absence of luminophore tracers from the surface sediment are the result of animal burrowing and feeding ^81^. Sediment cores were collected from these spots, and sliced into 2 cm increments as five subsamples and pooled from individual buckets.

### Luminophore recovery

Subsamples were stirred for 1 h and digested by 15% H_2_O_2_ solution for 1 week in clean glass beakers to remove organic matter ^82^. The digested subsamples were carefully rinsed through distilled water, placed in dust-free containers (70 ml), and freeze-dried for 72 h. Freeze-dried subsamples were weighed and homogenized. Visible shell fragments were picked out from the containers. A cohesive tape (size: 4.0 cm × 4.2 cm, transparent) was vertically inserted to the container and vortexed for 60 s (800 rpm) to homogenize all particles on the tape. The tape covered by luminophores and sediment particles was removed from the container and preserved in petri dishes, and the remaining sediment was re-weighed. The difference on the sample weight was the weight of the particles that attached on the cohesive tape (g). Triplicated tape samples were extracted from each container.

The amount of the luminophores spreads through sediment section (0–2 cm, 2–4 cm, 4–6 cm, 6–8 cm and 8–10 cm depths) reflects the vertical sediment mixing by bioturbation for the luminophore particle^83, 84^. Luminophore particles in the tape samples were photographed in a UV light chamber (34 × 26 × 30 cm), with four installed ultraviolet (UV) lights at top corners to deliver consistent illumination. Tape samples were horizontally laid on the bench within the chamber, under a 12 cm distance from camera lens (48-mega pixels, 25× zoom-in). Sample photographs (3024 × 4032 pixels) were binarized as black and white (0: pixels in black areas, 1: pixels in florescent areas) and luminophore particles were calculated by Image J 1.53a (Plugins, Blob labeler). The profiles of luminophore tracers from the laboratory test represents the sediment reworking by bioturbators interacting with MP (F_24,180_ = 39.037, p = 0.000, 3-way ANOVA test, See Table S1, Supplementary); These observations were fitting to the deterministic bioturbation model (details as below).

### Derived D_b_ by fitting sediment reworking profiles

The observed profiles of luminophore particles from the experiment were fitted to a deterministic bioturbation model to derived the bioturbation coefficients D_b_^77, 85^. This method has been applied in previous studies^16, 81^.

The predicted luminophore transportation at each depth was calculated as:

1 $$Lum\left( x \right) \, = \, \left( {\frac{{1}}{{\sqrt {\pi D_{b} t} }}} \right)e^{{ - x^{{2}} /{4}D_{b} t}}$$

where:

$$\frac{\partial Lum(x)}{{\partial x}} = 0,\quad when\quad x = 0$$

$$Lum(x \to \infty , \, t) = 0;$$

**Figure 6:**
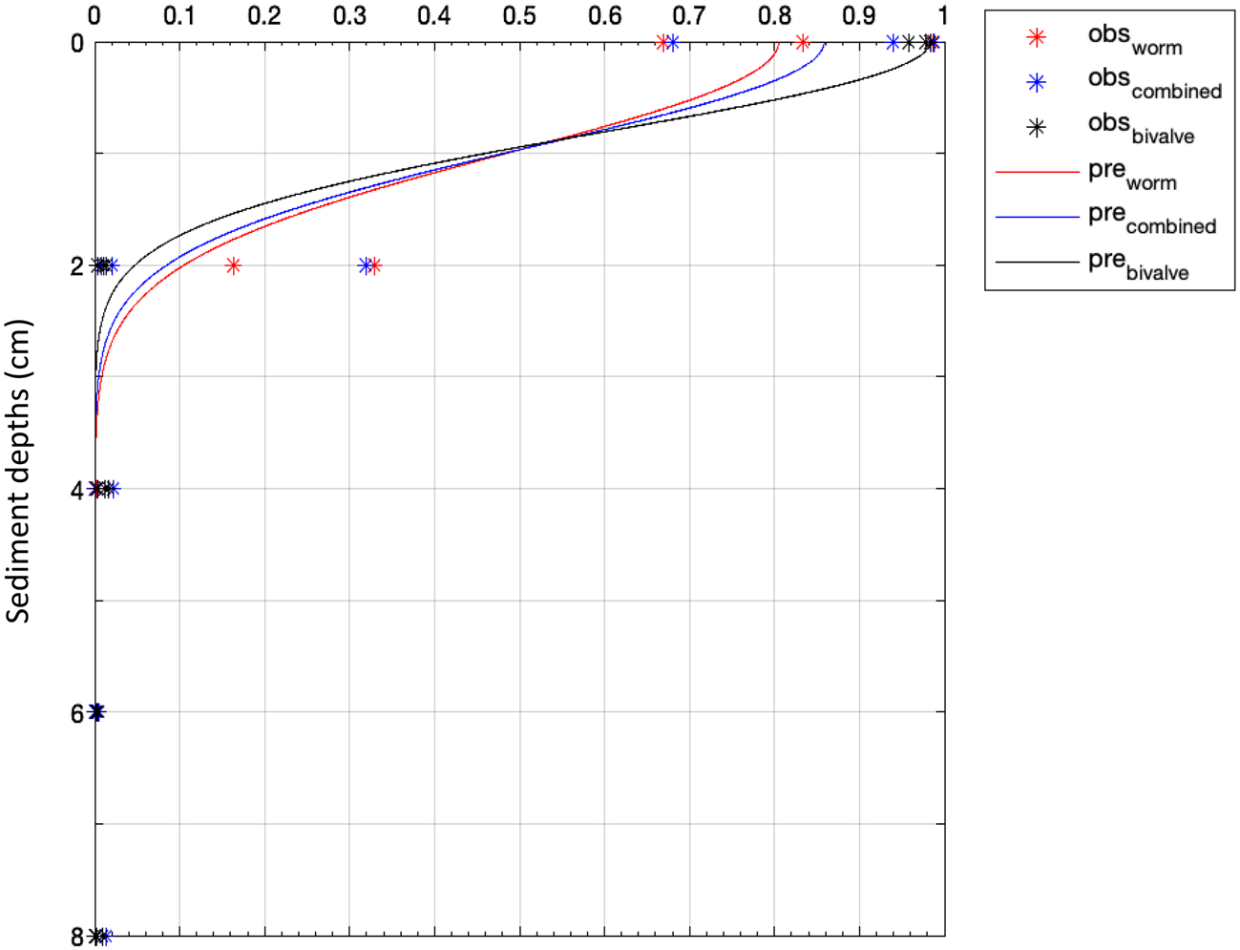
Examples of fitting the observational luminphore profiles (obs_i) to the prediction (pre_i) from bioturbation model to derive the value of D_b_ from maldanid worm, combined and tellinid bivalve groups in control. Other profiles from MP treatments are in Fig. S1, supplementary.

D_b_ is assumed to be consistent in time; t is the duration of the experimental days, 21 days; x is the sediment layers 0–2, 2–4, 4–6, 6–8, 8–10 cm (nominal depths: 0, 2, 4, 6, 8 cm); Lum(x) is the luminophore particles at depth *x*; D_b_ was derived from a convergent iteration and the weighted regression of least-squares comparison between the observational (*obs_i*) and predicted luminophore particle (*pred_i*) profiles (see profile example in Fig. 6) using the least square non-linear function (LSQNONLIN, Matlab, 2021b).

The iteration continued until observational data has minimum distance to prediction, resulting in the smallest residual values.

2 $${\text{Residuals }} = \sum\nolimits_{i = 1}^{n} {\frac{{\left( {obs\_i - pred\_i} \right)^{2} }}{{obs_{i} + 1}}}$$

Residual outputs have shown the fit of the predicted values to the observational data (see Fig. S2).

### Estimated impacts on ecosystem functions by fitting D_b_

The simplified transport-reaction model^34^ was applied to evaluate the ecosystem functioning associated to bioturbation in MP-contaminated system (parameters and constant values as shown in Table S2). We set a depth of 2 cm sediment as the boundary of oxic zone for aerobic mineralization (Z_o2_) which is in line to a common range of redox potential layer in the sediment^86^. OM burial was considered negligible due to the dominance of bioturbation^34^.

The profile of OM concentration along sediment depth (C(x), mmol OM g^−1^):

3 $$C\left( x \right) \, = \, C^{0} e^{{\left( { - x/z} \right)}} ;$$

where Z is the effective zone of bioturbation:

4 $${\text{Z }} = \frac{{2D_{b} }}{{ - \omega + \sqrt {\omega^{2} + 4 D_{b} k}}};$$

And the estimated OM concentration (C^0^_,_ mmol OM g^−1^) initially received from sediment–water interface (SWI) is:

5 $$C^{0} = \frac{2F\_OM}{{\rho (1 - \phi )[\omega + \sqrt {\omega^{2} + 4D_{b} k]} }};$$

The aerobic mineralization rate (R_(oxic_Db),_ mmol OM cm^−2^ year^−1^) at oxic zone:

6 $$R\left( {oxic\_D_{b} } \right) \, = \, Z_{o2} k*\rho (1 - \phi )*C_{avg} (D_{b} ) = Z_{o2} k\frac{F\_OM}{{D_{b} k}};$$

where C_avg_(D_b_) is the approximate average OM concentration in the oxic zone when D_b_ dominants the advective transport (ω = 0):

7 $$C_{avg} \left( {D_{b} } \right) \, = \frac{F\_OM}{{\rho (1 - \phi )\sqrt {D_{b} k} }};$$

The oxygen consumption rates (mmol cm^−2^ year^−1^) for aerobic mineralization:

8 $$F_{\_o2} \approx \, R\left( {oxic\_D_{b} } \right);$$

The fraction of aerobic mineralization (β) accounting to OM degradation:

9 $$\beta = \frac{{R_{(oxic\_Db)} }}{F\_OM} = \frac{{Z_{O2} k\frac{{F_{OM} }}{{\sqrt {D_{b} k} }}}}{F\_OM} = Z_{O2} *\sqrt {\frac{k}{{D_{b} }}} ;$$

The fraction of OM fluxes for reduction processes that can enhance denitrification (R_(N)_) :

10 $$R_{(N)} \cong F\_OM \, {-} \, R_{{\left( {oxic\_D_{b} } \right) \, }} = \, F\_OM \, * \, (1 - \beta )$$

F__OM_ is a fixed organic matter flux delivered to SWI as a boundary condition, average 0.6 mmol cm^−2^ year^−1^ in coastal environment; *k* is the organic matter decay constant (year^−1^), k = 0.1; ω is the advective velocity for solids and solutes, 0.1 cm year^−1^; ρ is the sediment density, 2.55 g cm^−3^; ϕ is the porosity, 0.35.

### Supplementary Information

Supplementary Information.

## Data Availability

Supplementary contains all relevant data as attached.
