# Supplementary material for: Modelled broad-scale shifts on seafloor ecosystem functioning due to microplastic impacts on bioturbation
Source: Sci Rep. 2023 Oct 10;13:17121. doi: 10.1038/s41598-023-44425-8 (PMC10564913; doi:10.1038/s41598-023-44425-8)
Supplement: Supplementary file 1 — Supplementary Information. [file 41598_2023_44425_MOESM1_ESM.docx]

**Supplementary**

| **Table S1 3-way ANOVA test on observational data** | | | | | |
| --- | --- | --- | --- | --- | --- |
| Dependent Variable: luminophore (NO. cm^-2^) | | | | | |
| Source | Type III Sum of Squares | df | Mean Square | F | Sig. |
| Model | 664173.678^a^ | 60 | 11069.561 | 1377.424 | 0.000 |
| Species | 324.848 | 2 | 162.424 | 20.211 | 0.000 |
| MP | 3050.043 | 3 | 1016.681 | 126.509 | 0.000 |
| depth | 489632.064 | 4 | 122408.016 | 15231.65 | 0.000 |
| Species * MP | 1752.47 | 6 | 292.078 | 36.344 | 0.000 |
| Species * depth | 1378.606 | 8 | 172.326 | 21.443 | 0.000 |
| MP * depth | 8763.659 | 12 | 730.305 | 90.874 | 0.000 |
| Species * MP * depth | 7529.271 | 24 | 313.72 | 39.037 | 0.000 |
| Error | 964.371 | 120 | 8.036 |  |  |
| Total | 665138.049 | 180 |  |  |  |
| R Squared = .999 (Adjusted R Squared = .998)  Performed in SPSS (IBM version 25) | |  |  |  |  |

| a | Control (0) | b | Low (0.0002 g cm^-2^) | 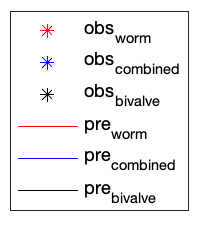 |
| --- | --- | --- | --- | --- |
| Ratio of penetrated luminophores | | Ratio of penetrated luminophores | |  |
| Sediment depths (cm) | 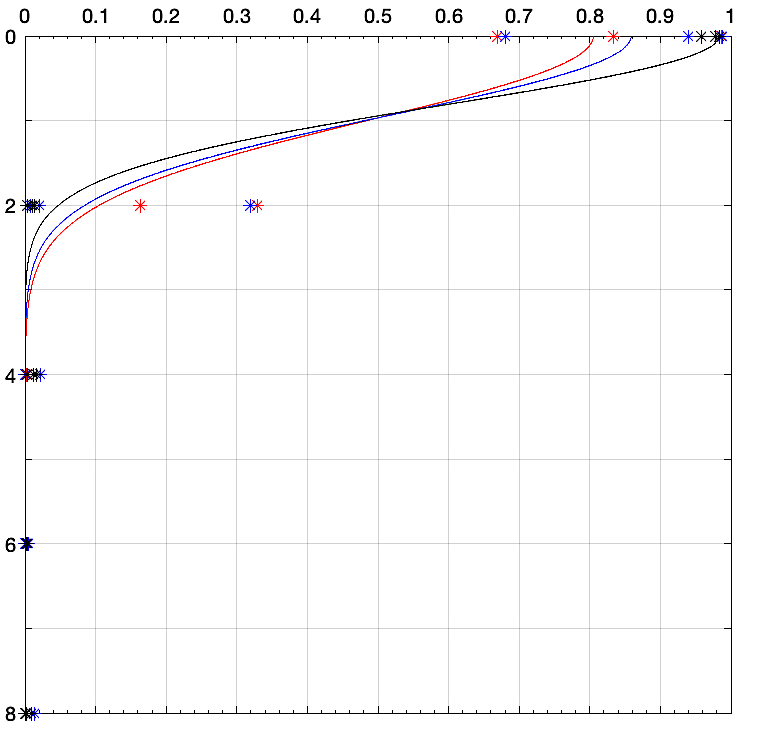 | 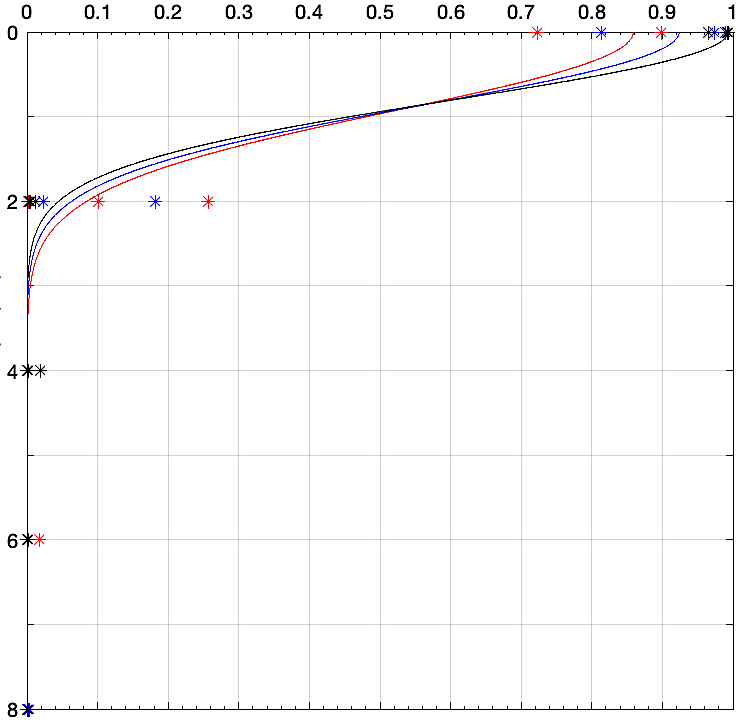 | |  |

| c. | Medium (0.002 g cm^-2^) | d | High (0.02 g cm^-2^) | 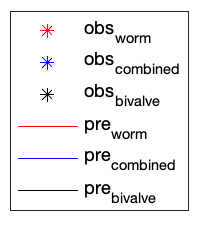 |
| --- | --- | --- | --- | --- |
| Ratio of penetrated luminophores | | Ratio of penetrated luminophores | |  |
| Sediment depths (cm) | 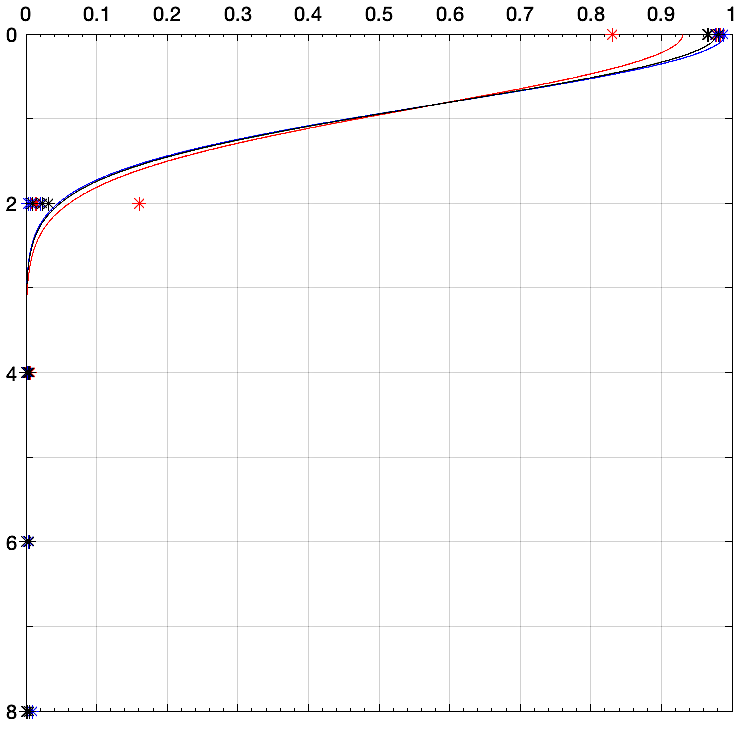 | 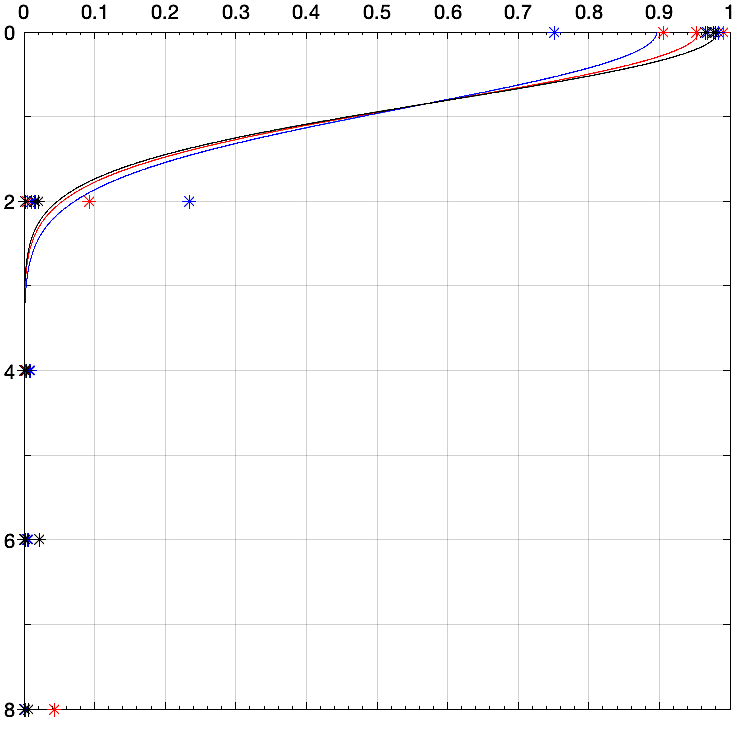 | |  |
| Figure S1 Fitting observations to bioturbation model in worm, combined and bivalve system when microplastic concentration increased from 0 (control) to 0.0002 (low), 0.002(medium), 0.02 (high) g cm^-2^ levels. Obs_i: observational data, Pre_i: prediction from bioturbation model. | | | | |

Figure S2 Residuals of from prediction in bioturbation model at each sediment depth 0-2cm (top1), 2-4cm(top2), 4-6cm(middle), 6-8cm (bottom2), 8-10cm (bottom1).

| **Table S2 Summarized parameter in transport-reaction model modified from Meysman et al., 2013** | | | |
| --- | --- | --- | --- |
| **Geometrical and environmental condition** | | | |
| Sediment length* | L | 30 | cm |
| Porosity* | 𝜙 | 0.35 | - |
| Solid-phase density* | 𝝆 | 2.55 | g cm^-3^ |
| Advective velocity(solutes and solids)* | $\omega$ | 0.1 | cm yr^-­1^ |
| **Reaction parameters** | | | |
| OM decay constant*  First-order rate constant | k | 0.1 | yr^-1^ |
| **Boundary parameters** | | | |
| Fixed OM fluxes at SWI * | F^0^_OM_ | 0.6 | mmol C cm^-2^ yr^-1^ |
| Total O_2_ consumption for OM degradation* | F_o2_ | 0.6 | mmol cm^-2^ yr^-1^ |
| Fixed oxygenated zone | O_2__max | 2 | cm |
| The parameter from Meysman et al., (2013) | | | |

| 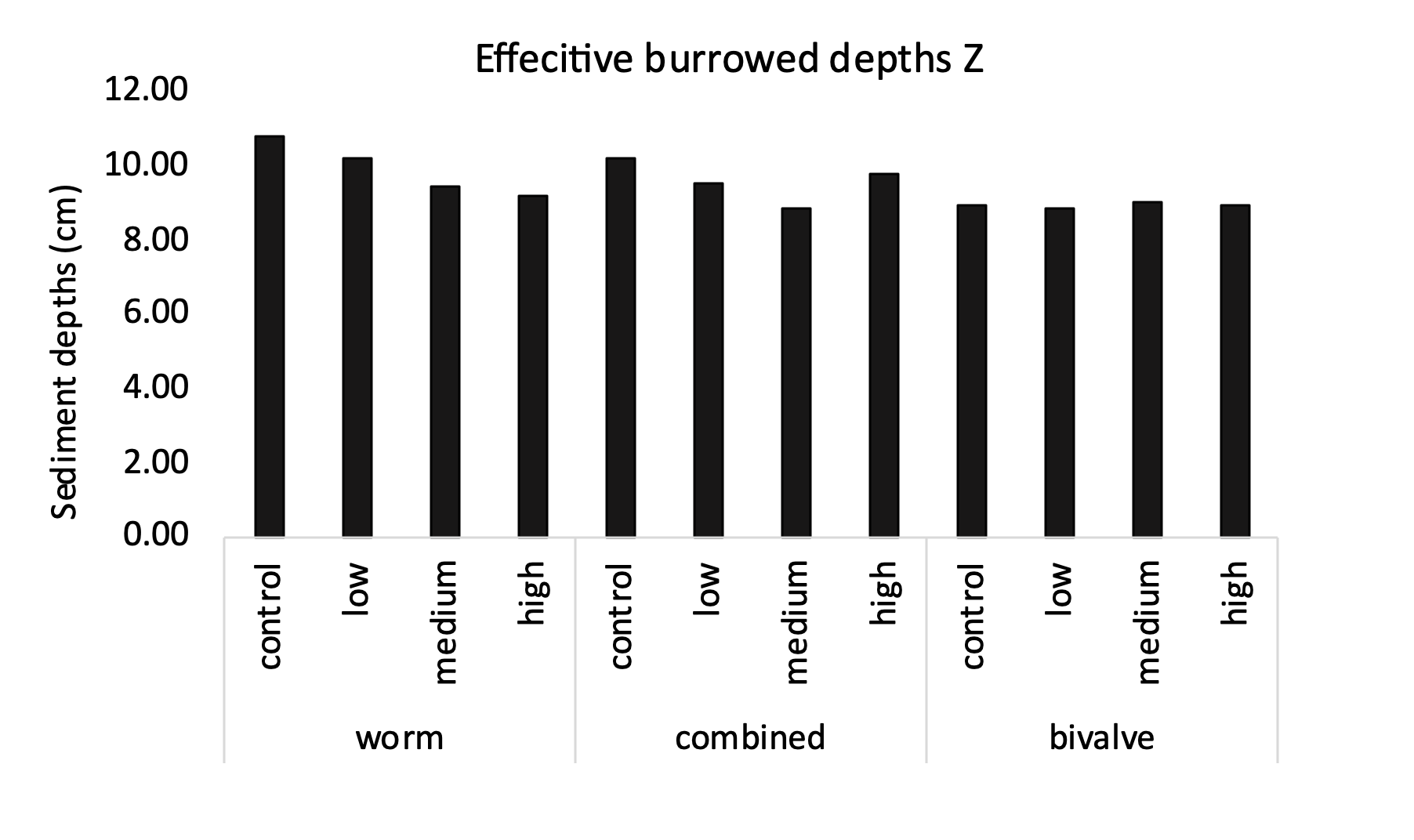 |
| --- |
| Figure S3 a. Effective burrowed depths Z in worm, combined and biavel groups control (0), low(0.0002 g cm^-2^), medium (0.002 g cm^-2^) and high (0.02 g cm^-2^) MP levels. |

| 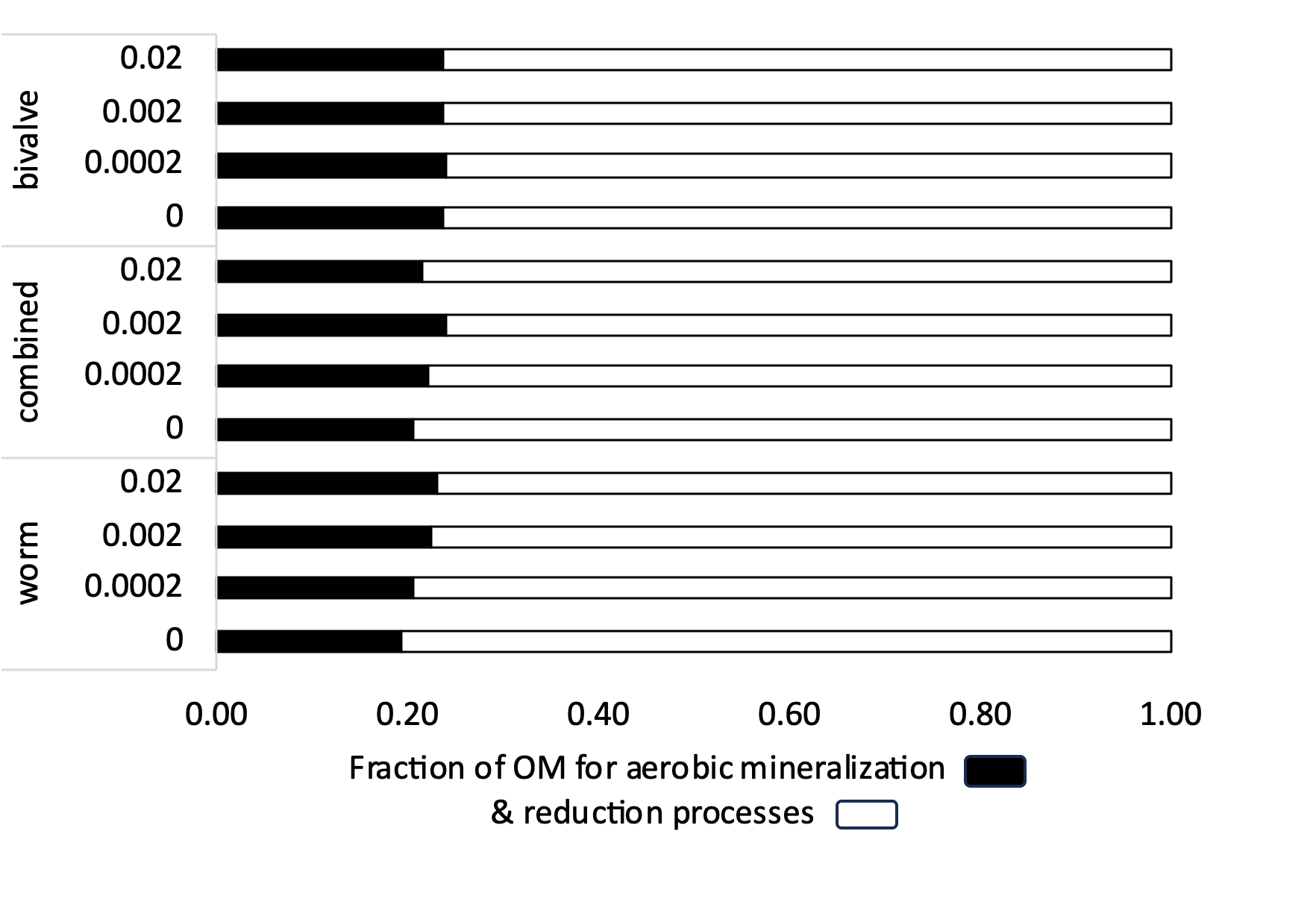 |
| --- |
| Figure S4 Fraction of the aerobic mineralization β and reduction processes (1- β) in worm, combined and bivalve groups when MP concentration increased from 0 to 0.0002, 0.002 and 0.02 g cm^-2^_._ |
